# Periestropause enhances hypoxia‐induced panic and microglial remodelling without altering respiratory control

**DOI:** 10.1111/jne.70254

**Published:** 2026-09-03

**Authors:** Angela Cristina Nicola, Luana Tenório‐Lopes, Luis Gustavo A. Patrone, Mariana Bernardes‐Ribeiro, Heloisa H. Vilela‐Costa, Daniele Gandra, Ana Clara Campideli‐Santana, Raphael E. Szawka, Kênia C. Bícego, Rita Cássia M. Dornelles, Luciane H. Gargaglioni

**Affiliations:** ^1^ Department of Animal Morphology and Physiology, School of Agricultural and Veterinary Sciences São Paulo State University (UNESP) Jaboticabal São Paulo Brazil; ^2^ Department of Physiology Institute of Bioscience, Sao Paulo State University‐UNESP Botucatu São Paulo Brazil; ^3^ Department of Physiology and Biophysics Institute of Biological Sciences, Federal University of Minas Gerais (UFMG) Belo Horizonte Minas Gerais Brazil; ^4^ Department of Biochemistry, Pharmacology and Physiology Institute of Biological and Natural Sciences, Federal University of Triângulo Mineiro Uberaba Minas Gerais Brazil; ^5^ Multicenter Graduate Program in Physiological Sciences (SBFis/UNESP) Araçatuba São Paulo Brazil; ^6^ Department of Basic Sciences, School of Dentistry São Paulo State University (UNESP) Araçatuba São Paulo Brazil

**Keywords:** ageing, hypercapnia, hypoxia, locus coeruleus, menopause

## Abstract

Perimenopause is characterised by irregular reproductive cycles and hormonal fluctuations, notably decreased estradiol (E_2_), which are linked to increased susceptibility to panic disorder and neuroinflammation. We examined whether panic‐related respiratory and behavioural responses to respiratory challenges vary across reproductive stages. Female rats with regular estrous cycles (RC), representing reproductive cyclicity, and rats in periestropause (PE), a stage similar to perimenopause, were exposed to normoxia, hypoxia, and hypercapnia. Ventilation (V̇_E_) and metabolism (V̇O_2_) were measured during diestrus in RC and PE rats. Additionally, to measure levels of neuroinflammation, microglial reactivity in the Locus coeruleus (LC) in response to hypoxia‐induced panic‐like attacks was assessed. Under normoxia, there were no differences in V̇_E_ or V̇O_2_ between groups. Hypercapnia increased V̇_E_ in both groups by elevating respiratory frequency (f_R_) and tidal volume (V_T_), without changing body temperature (Tb). Hypoxia also raised V̇_E_ in both groups, primarily driven by increased f_R_, and hypoxia‐induced hypothermia occurred in RC and PE females. Both hypercapnia and hypoxia elevated the V̇_E_/V̇O_2_ ratio. Exposure to 7% O_2_ heightened panic‐like behaviours in PE rats. Hypoxia increased LC microglial number, density, and mean soma size, while decreasing arborization area. PE rats also showed a higher microglial morphological index and a shorter nearest neighbour distance (NND). Jump frequency and freezing duration positively correlated with the morphological index and negatively with arborization area. These findings suggest that respiratory regulation remains intact, but behavioural and neuroimmune responses are amplified during the transition to estropause, heightening vulnerability to panic and anxiety‐related disorders in ageing females.

## 1 INTRODUCTION

Biological ageing presents unique challenges, especially in female organisms, which are highly susceptible to systemic changes caused by hormonal fluctuations associated with the end of reproductive cycles.^1^, ^2^ During this process, estrous cycle regularity gradually shifts to irregularity, acyclicity, and complete loss of fertility,^3^ due to neuronal changes in central reproductive regions that affect the hypothalamic–pituitary‐gonadal (HPG) axis.^4^, ^5^, ^6^ These neuroendocrine changes, along with the decline in ovarian follicle reserves and steroid hormone production, orchestrate the progressive disruption of reproductive cycles, ultimately leading to menopause.^5^, ^7^ Moreover, age‐related impairments in the suprachiasmatic nucleus (SCN) can lead to misalignment of circadian and hormonal rhythms, exacerbating irregular cycle patterns and reproductive ageing.^8^ Overall, the interaction of neuronal changes in the central nervous system, altered feedback within the HPG axis, and circadian disruption drives the transition from regular cycles to infertility during reproductive ageing.^8^

The perimenopausal phase is marked by notable hormonal instability, particularly a decline in circulating 17β‐estradiol (E_2_) levels. This decline is associated with a higher risk of mood disorders like depression and anxiety.^9^ Anxiety disorders, including panic disorder (PD), represent a significant yet under‐investigated aspect of the menopausal transition in both humans and animal models.^10^ The prevalence of PD during perimenopause is notably influenced by fluctuations in female reproductive cycles, with symptoms often worsening as menopause approaches.^11^ Women aged 40–49 years exhibit a PD prevalence rate of about 3.3%, with hormonal changes, especially the reduction in E_2_, playing a key role in this increase.^10^ Anxiety and panic symptoms tend to peak during this phase due to these hormonal shifts, which destabilise neuroendocrine and neurotransmitter systems involved in anxiety regulation.

Panic attacks (PAs) have been hypothesised to result from a neuroendocrine, autonomic, and behavioural response triggered by a false suffocation alarm due to respiratory chemoreceptor hypersensitivity to elevated CO_2_ or reduced O_2_ levels.^12^, ^13^, ^14^ In this context, hypoxia not only acts as a powerful trigger for increased ventilation, primarily through the activation of the carotid bodies, but also induces a strong panic‐like response in both male and female rats^15^ and in humans.^16^, ^17^ This panic‐like reaction is mediated by neural pathways that overlap with those involved in panic and anxiety states in humans.^18^, ^19^ Unlike mice, exposure of rats to 7% O_2_, but not to severe hypercapnia, elicits defensive behaviours and endocrine changes that suggest the animals are experiencing a highly aversive situation, akin to a panic attack.^15^, ^20^ Therefore, hypoxia has a dual effect: it triggers physiological adaptation through increased respiration, and it provokes intense emotional and behavioural responses similar to those observed in rodent panicogenic responses.

The LC, a noradrenergic nucleus located in the brainstem, is highly sensitive to CO_2_/pH changes and plays a vital role in regulating ventilation^21^, ^22^ and in anxiety‐ and panic‐related responses.^23^ We have demonstrated that mice lacking noradrenergic neurons in the LC exhibited a markedly reduced jumping response and a prolonged freezing behaviour compared with control animals. These findings suggest that LC noradrenergic neurons facilitate the transition from passive defensive behaviours (freezing) to active escape responses (jumping) during CO_2_‐induced panic‐like states, supporting a key role for the LC in coordinating adaptive behavioural responses to panicogenic stimuli.^23^

LC modulates the reproductive axis primarily via noradrenergic projections to the arcuate nucleus, where it influences kisspeptin/KNDy neurons that function as the gonadotropin‐releasing hormone (GnRH) pulse generator^24^; this upstream control shapes downstream GnRH pulsatility and luteinising hormone secretion. In female mice, LC‐derived noradrenaline appears to act directly on arcuate kisspeptin neurons via β‐adrenergic receptors, hyperpolarising these cells and dampening pulse‐generator activity.^25^ Earlier work also showed that norepinephrine can directly suppress GnRH neuron excitability under some conditions.^26^ Thus, the LC is best viewed as a brainstem noradrenergic hub that conveys stress‐ and state‐dependent signals to the hypothalamic reproductive network rather than as a simple monosynaptic drive onto GnRH neurons.^24^

Studies on female rodents also show that the LC significantly impacts ovulatory cycles and that its neuronal activity changes with age.^5^ In humans, a recent study found stronger connectivity between the left LC and the executive control network in premenopausal women compared to men, which may relate to their higher anxiety symptom scores.^27^ Interestingly, no significant sex differences were observed for the right LC, suggesting that functional asymmetry may exist within the LC. As life expectancy increases, research on neurodegenerative diseases has grown, and the LC is especially vulnerable to these processes.^28^

The drop and fluctuations in E_2_ characteristic of perimenopause are also strongly linked to increased microglial activation and brain inflammation.^29^ Microglia, a type of immune cell in the central nervous system (CNS), express estrogen receptors,^30^ mainly the ERβ subtype,^31^ and are highly responsive to oxygen deprivation.^32^ When these receptors are activated, they suppress the secretion of pro‐inflammatory cytokines.^33^ This response is a key part of the anti‐inflammatory effects of estrogens in the CNS.^34^, ^35^

Considering the impact of ageing on physiological homeostasis and the unclear effect of E_2_ in modulating anxiety and panic‐related respiratory disturbances, this study aimed to (1) evaluate the ventilatory response to hypercapnic (7% CO_2_) and hypoxic (7% O_2_) challenges in rats with a regular estrous cycle (RC) and in rats in periestropause (PE); (2) assess panicogenic behavioural responses caused by hypoxia in RC and PE rats; and (3) explore how microglial cells in the LC respond to hypoxic stimuli in RC and PE rats.

## 2 MATERIALS AND METHODS

### 2.1 Animals

Experiments were conducted on female *Wistar* rats, including 4‐month‐old rats with a RC for physiological reference of reproductive cyclicity and evaluated in diestrus II, as well as 18‐month‐old rats in PE. The animals were selected according to the criteria previously described.^5^, ^36^ They had free access to water and food and were housed in a temperature‐controlled room (25°C ± 1°C) with a 12:12‐h light–dark cycle (lights on at 6:00 am). The study followed the guidelines of the National Council for the Control of Animal Experimentation (CONCEA, MCT, Brazil) and received approval from the local ethics committee (CEUA, FCAV‐UNESP Jaboticabal; Process n^o^. 8417/22). Ventilatory and behavioural experiments and blood sampling were performed between 9:00 am and 1:00 pm to minimise the influence of circadian and estrous cycle–related factors. All procedures were carried out at the Department of Animal Morphology and Physiology, Faculty of Agricultural and Veterinary Sciences (FCAV‐UNESP, Jaboticabal campus).

### 2.2 Assessment of stages of the estrous cycle

The animals underwent daily vaginal lavages to monitor the progression of the estrous cycle. These lavages were performed each day at 9 am to verify regularity over at least 15 days. The samples were examined by direct cytology under a light microscope. Only females exhibiting at least three consecutive regular cycles were included in the RC experiment group. Estrous cycle phases were determined based on the presence of the following predominant cell types: (1) proestrus: nucleated cells; (2) estrus: cornified epithelial cells; (3) metestrus or diestrus I: presence of nucleated, cornified epithelial cells and some leukocytes; and (4) diestrus II: leukocyte cells.^37^ Regarding the inclusion criteria for the PE group, only animals with irregular cycles in the persistent diestrus phase were considered, as the onset of reproductive senescence is characterised by this phase.^5^, ^38^

### 2.3 Body temperature (Tb)

Before conducting the ventilatory and metabolic experiments, the rats underwent surgery to implant an abdominal temperature sensor‐datalogger (12 mm; APT12 PIT Tag, Biomark, USA). All surgical procedures were performed under anaesthesia with isoflurane (5% for induction and 1% for maintenance), along with postoperative antibiotic prophylaxis (2.5 mg/kg, s.c.; enrofloxacin, Flotril, Schering‐Plough, São Paulo, Brazil) and analgesia (10 mg/kg, s.c.; flunixin meglumine, Banamine, Schering‐Plough, São Paulo, Brazil). The abdominal region was shaved, followed by antisepsis with 70% alcohol and chlorhexidine degerming, and the sensor was inserted into the abdominal cavity through a midline laparotomy. Subsequently, the region was sutured. After surgery, the animals were housed in boxes with ad libitum access to water and food and kept in a room with controlled ambient temperature, humidity, and light. After surgery, the estrous cycle was evaluated daily, and following 7 days of recovery, the animals were subjected to the experimental protocol.

### 2.4 Pulmonary ventilation (V̇_E_) and oxygen consumption (V̇O_2_)

**FIGURE 1:**
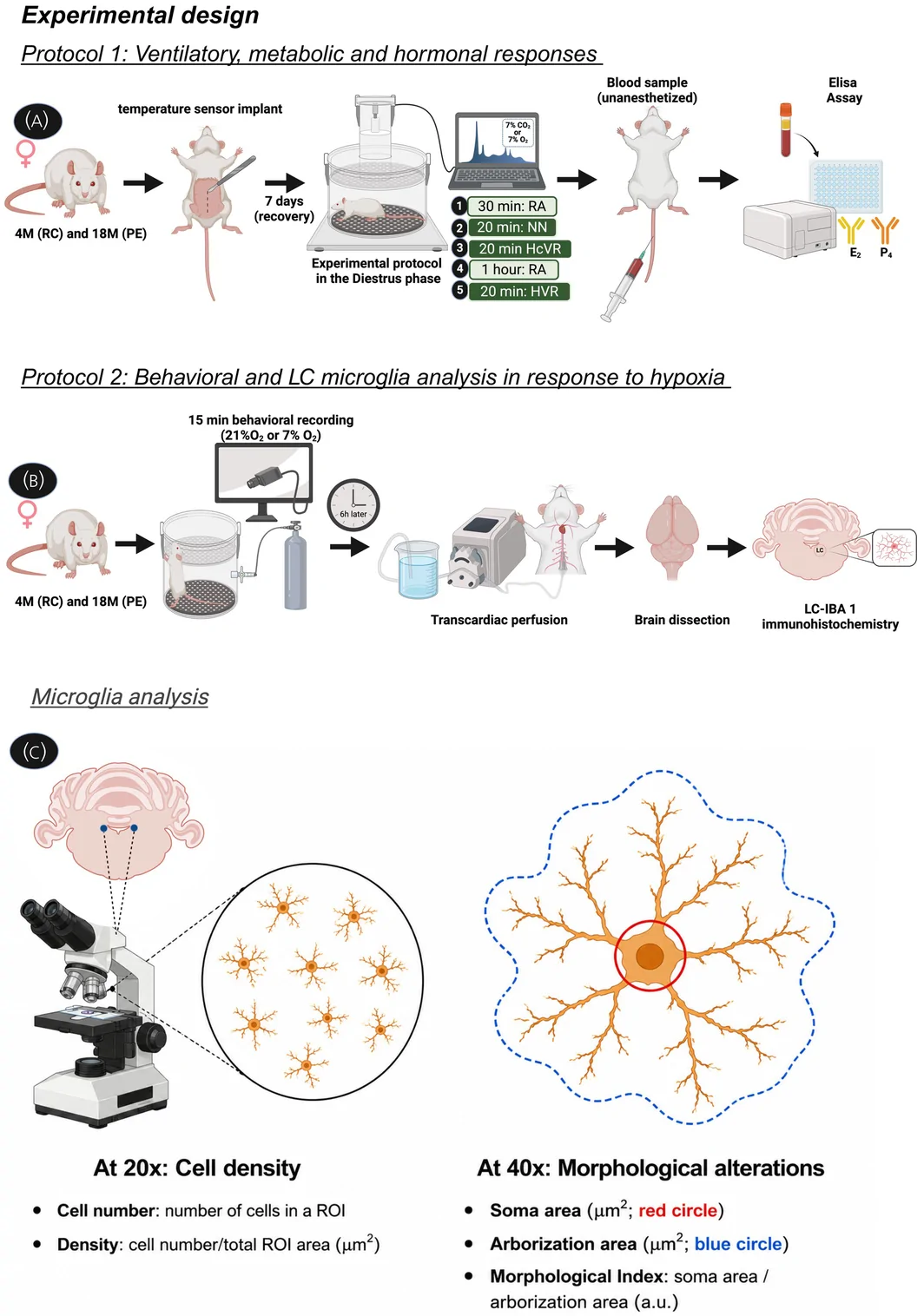
Experimental design of the study. (A) Whole‐body plethysmography protocol for analysing ventilation, metabolism, and hormonal assays. (B) Protocol for behavioural and microglial cell analyses of the LC in response to panicogenic stimulation with 7% O_2_. (C) Morphometric analysis of microglia morphology. Schematic illustration of the measurements performed. The microglial soma is outlined for quantification of soma area (or soma size), and the ramification is shown to illustrate the parameters used to assess arborization. HcVR, hypercanic ventilatory response; HVR, hypoxic ventilatory response; NN, normoxia normocapnia; RA, room air; ROI, region of interest.

In a group of rats (RC in diestrus II, *N* = 6–8; PE, *N* = 7), measurements of V̇_E_ and V̇O_2_ were performed under normocapnic, normoxic conditions, hypercapnia, and hypoxia (Figure 1A). To this end, animals were placed in a 5 L plexiglass chamber (Bonther, SP, Brazil) and allowed to move freely while the chamber was flushed with humidified room air. V̇_E_ was measured using a whole‐body plethysmography‐closed system.^21^, ^39^ The air inlet and outlet flows were 2 L/min, measured by a flowmeter (‘Mass Flow System’ – MFS, Sable Systems International, Inc., Las Vegas, NV). Signals were detected by a differential pressure transducer (TSD 160A, Biopac Systems, Santa Barbara, CA), collected via an amplifier (DA 100C, Biopac Systems), and digitised through an analogue‐to‐digital converter connected to a computer with acquisition software (AcqKnowledge MP 100, BioPac Systems, Inc., Santa Barbara, CA). *f*_R_ and *V*_T_ were measured, with *V*_T_ calculated using an appropriate formula^39^, ^40^:

$$V_{T}=\mathrm{VK}\times \left(\mathrm{PT}/\mathrm{PK}\right)\times \mathrm{TC}\times \left(\mathrm{PB}-\mathrm{PC}\right)/\mathrm{TC}\times \left(\mathrm{PB}-\mathrm{PC}\right)–\mathrm{TA}\times \left(\mathrm{PB}-\mathrm{PR}\right)$$

PT represents the pressure deflection corresponding to each *V*_T_, PK corresponds to the pressure deflection associated with the injection of the calibration volume (VK), TA indicates the air temperature inside the animal chamber, and PC is the water vapour pressure in the animal chamber. At the same time, Tb is the body temperature, and PR indicates the water vapour pressure at Tb. The PC and PR were calculated using Dejours's table.^41^ *V*_E_ and *V*_T_ values were presented under standard conditions, accounting for ambient barometric pressure conditions at Tb and saturated with water vapour (BTPS), assuming expired air was fully saturated with water vapour because a layer of water was placed at the bottom of the animal's chamber.

O_2_ consumption (V̇O_2_) was obtained using the indirect calorimetry method as previously described.^39^ The CO_2_ was not analysed or scrubbed; then, aiming at a better metabolic rate measurement, the V̇O2 was calculated using the following equation^42^:

$$\dot{V}O_{2}=\left[\mathrm{Fre}\left(\mathrm{Fi}O_{2}-\mathrm{Fe}O_{2}\right)\right]/\left[1-\mathrm{Fi}O_{2}\left(1-\mathrm{RQ}\right)\right]$$

where FRe is the end flow rate of air through the chamber, FiO_2_ is the inlet O_2_ fraction, FeO_2_ is the end O_2_ fraction, and RQ is the respiratory quotient (considered to be 0.85). The V̇O_2_ was divided by body mass, and values were presented as STPD (standard conditions of temperature, pressure and dry air).

The rats were placed in the experimental chamber for a 30‐min habituation period with room air. After this period, two ventilatory and metabolic measurements were taken under conditions of normocapnic normoxia, with a 10‐min interval between each. For V̇_E_ measurements, the chamber was fully sealed for approximately 2 min. Subsequently, the animal was exposed to a hypercapnic gas mixture (7% CO_2_, 21% O_2_ and N_2_ balance) for 20 min, and V̇_E_, V̇O_2_ and Tb were measured at the end of this exposure. After a 60‐min recovery period in normocapnic normoxic air, the animals underwent 20 min of hypoxia (7% O_2_ and N_2_ balance). A final ventilatory and metabolic measurement was then performed at the end of the hypoxic exposure. The respiratory and metabolic parameters were analysed from 2 min of recordings during room air, hypercapnia and hypoxia.

### 2.5 Blood collection for plasma hormone assays

After the hypoxic challenge and just before euthanasia, a blood sample of approximately 1 mL was taken from the tail of anesthetized rats into an Eppendorf tube heparinized with a diluted heparin solution (0.15 mL of sodium heparin at 5000 IU/mL in 3 mL of 0.9% NaCl) to measure the plasma levels of P_4_ and E_2_ of RC and PE rats (*N* = 11–15 per group after exposure to normoxia or hypoxia). The plasma was separated by centrifugation at 3000 rpm for 10 min at 4°C and then stored at −80°C. Plasma E_2_ and P_4_ levels were determined using enzyme‐linked immunosorbent assay (ELISA) kits (E_2_: Ref 4925‐300A, lot EIA‐49K6J1; P_4_: Ref 4825‐300A, lot EIA‐48K2J1; Monobind Inc., CA), following the manufacturer's instructions. The lower detection limits for E_2_ were 8.2 pg/mL, and for P_4_ were 0.105 ng/mL. The intra‐assay coefficients of variation were 7.5% and 5.1%, respectively. Both assays were validated using samples from diestrus, proestrus, and OVX rats. E_2_ is expressed in pg/mL, P4 in ng/mL.

### 2.6 Panicogenic stimulus

In another group of rats (RC in diestrus, *N* = 7; PE, *N* = 8), behavioural analysis was conducted during a hypoxic challenge (Figure 1B). Panicogenic behaviour was induced using 7% O_2_.^15^, ^43^ Initially, the animals were acclimated for 30 min, followed by 10 min of behavioural recording under normocapnic normoxic conditions. Then, N_2_ was ventilated into the chamber at 2 L/min for approximately 2 min, achieving an [O_2_] of 7.6%. Immediately afterwards, a mixture containing 7% O_2_ was connected to the chamber to maintain this concentration for 15 min. To assess panicogenic responses, experiments were recorded with a camera (Logitech, USA) positioned above the chamber to capture the animals' behaviour during the entire time considered. The escape behaviours analysed included the number of jumps, the duration of running (horizontal escape), and freezing (a complete lack of movement except for breathing). Rearing (escape behaviour) and grooming behaviour (a behavioural marker of moderate stress and anxiety)^44^, ^45^ were also evaluated. The analysis was performed offline by a trained observer blinded to the animals' conditions. Behavioural analyses were performed on distinct groups of animals under normocapnic and hypoxic conditions, owing to the requirement for euthanasia 6 h post‐stimulus.

Behaviours were scored according to predefined operational criteria.^15^, ^43^ Defensive responses included the active responses of jumping and horizontal running, as well as the passive response of freezing. Jumping (vertical escape) was recorded as each episode in which the animal propelled all four paws off the floor towards the chamber walls or lid, regardless of whether contact was made with the surface. Running (horizontal escape) was recorded as rapid locomotion across the chamber floor, involving coordinated, high‐speed movement of all four limbs. Freezing was defined as the complete absence of body and head movements, except those required for respiration, lasting at least 3 s. Exploratory behaviour was assessed by rearing, recorded whenever the animal adopted a vertical posture by lifting both forelimbs from the floor while standing on the hindlimbs, with or without contact between the forepaws and the chamber wall. Self‐maintenance behaviour was assessed by grooming, quantified as the cumulative duration of stereotyped self‐cleaning movements directed towards the face, head, body, forelimbs, hindlimbs, or tail.

### 2.7 IBA‐1 immunohistochemistry and microglia analysis

Six hours after the induction of the panicogenic stimulus via hypoxic challenge, the same rats used in the previous protocol were deeply anaesthetised with ketamine (ketamine hydrochloride, Syntec® 80 mg/kg, pc/ip) and xylazine (xylazine hydrochloride, Syntec® 40 mg/kg, pc/ip), then transcardially perfused with phosphate‐buffered saline (PBS), followed by ice‐cold 4% paraformaldehyde. After cryoprotection in 30% sucrose, serial coronal brain sections of 30 μm were cut in four series representing the anteroposterior length of the LC. IBA‐1 immunohistochemistry was performed on free‐floating sections. Briefly, the sections were incubated overnight at 4°C with rabbit polyclonal IBA‐1 antibody (1:250, Wako, Osaka, Japan). They were then incubated with biotinylated anti‐rabbit IgG (1:400, Vector) in blocking solution for 3 h, followed by incubation with avidin‐biotin complex (ABC, Vector Laboratories) at 1:600 for 2 h. After washing in 0.01 M PBS, the sections were first treated with 1% H_2_O_2_ for 20 min, then with the DAB chromogen tablet (3,3’‐diaminobenzidine SIGMAFAST™ DAB) for 5 min. The reaction was halted by washing the sections four times in 0.01 M PBS for 5 min each. The sections were mounted on gelatin‐coated slides, sequentially dehydrated in increasing concentrations of ethyl alcohol (70%, 80%, 95%, 100%), and cleared in xylene before being coverslipped with Entellan (Merck®).

To investigate the reactivity of microglia in our region of interest (ROI), the LC, representative sections from five animals in the RC group and six from PE females at similar anatomical levels were analysed (Bregma −9.96 mm)^46^ (Figure 1C). Bilateral photomicrographs at 20× magnification were used to evaluate microglial density, while 40× magnification assessed microglia morphology (cell body and arborization areas). Both the left and right sides of the LC were examined for all parameters using ImageJ (NIH, U.S. National Institutes of Health).

First, the number of microglial cells in the LC region was quantified, then the cell density was calculated as the ratio of the total number of cells to the total area of the LC.^47^, ^48^ To analyse morphology, a manual drawing outlined each cell, including arborizations and cell body areas (Figure 1C). This process was repeated for at least 10 neighbouring cells. From these average values, the morphological index (soma area/arborization) was determined. This index indicates changes in microglial shape,^49^ reflecting changes in cellular ramification and complexity. Higher index values indicate a more ramified, surveillant morphology, characterised by a small soma and long, highly branched processes, whereas lower values indicate a shift toward a more reactive morphology, characterised by an enlarged soma, shorter and fewer processes, reduced branching complexity, and a more amoeboid appearance. As microglial activation occurs along a continuum, this index should be interpreted as an objective measure of morphological changes rather than a classification into discrete phenotypic states. Finally, to measure nearest‐neighbour distances, we used images at 40× magnification and the plugin ‘NND’ in ImageJ. In the region of interest (ROI; the right and left LC), each microglia from the 10 microglia cells previously selected (to investigate arborization and cell body areas) was compared with its nearest neighbouring microglia^48^; for step‐by‐step analysis, please see Ibanez et al.^50^ The NND is an index of cell dispersion, indicating microglial cell motility.^47^, ^48^ In all analyses, the experimenter was blinded to the identities of the animals and treatments.

### 2.8 Data analysis

The statistical analyses for all data, except behavioural data, were conducted using SigmaPlot (version 14.5). Data were evaluated for compliance with the assumptions of homogeneity of variances and normality of Studentized errors. Body mass, E_2_ and P_4_ plasma concentrations were analysed using an unpaired *t*‐test. A two‐way repeated‐measures ANOVA, followed by Tukey's test, was performed on ventilatory, metabolic and microglial parameters including age, gas condition, and their interaction as factors.

Behavioural variables were analysed using a generalised linear model (GLM) with a negative binomial distribution because they consisted of overdispersed count data with a high frequency of zero observations. The large number of zeros was expected, as panic‐like defensive behaviours (e.g., running, jumping and escape) are rarely expressed under room air conditions and are primarily elicited by hypoxic exposure. The model included Age, Exposure, and their interaction as fixed effects. Statistical significance was assessed using Type III analysis of deviance (Wald χ^2^ tests), followed by Šídák‐adjusted pairwise comparisons of estimated marginal means when appropriate. These analyses were performed on the R platform (version 4.2.3, R Development Core Team, 2023) in RStudio (version 2023.12.0). Spearman's correlation test was used to assess associations between behavioural data and microglial parameters. Data are presented as mean ± SD, with a significance level of 5% (*p* < .05) for all tests.

## 3 RESULTS

All statistical information is shown in Tables S1–S5.

### 3.1 Body mass, estrous cycle regularity, and hormonal concentration

**FIGURE 2:**
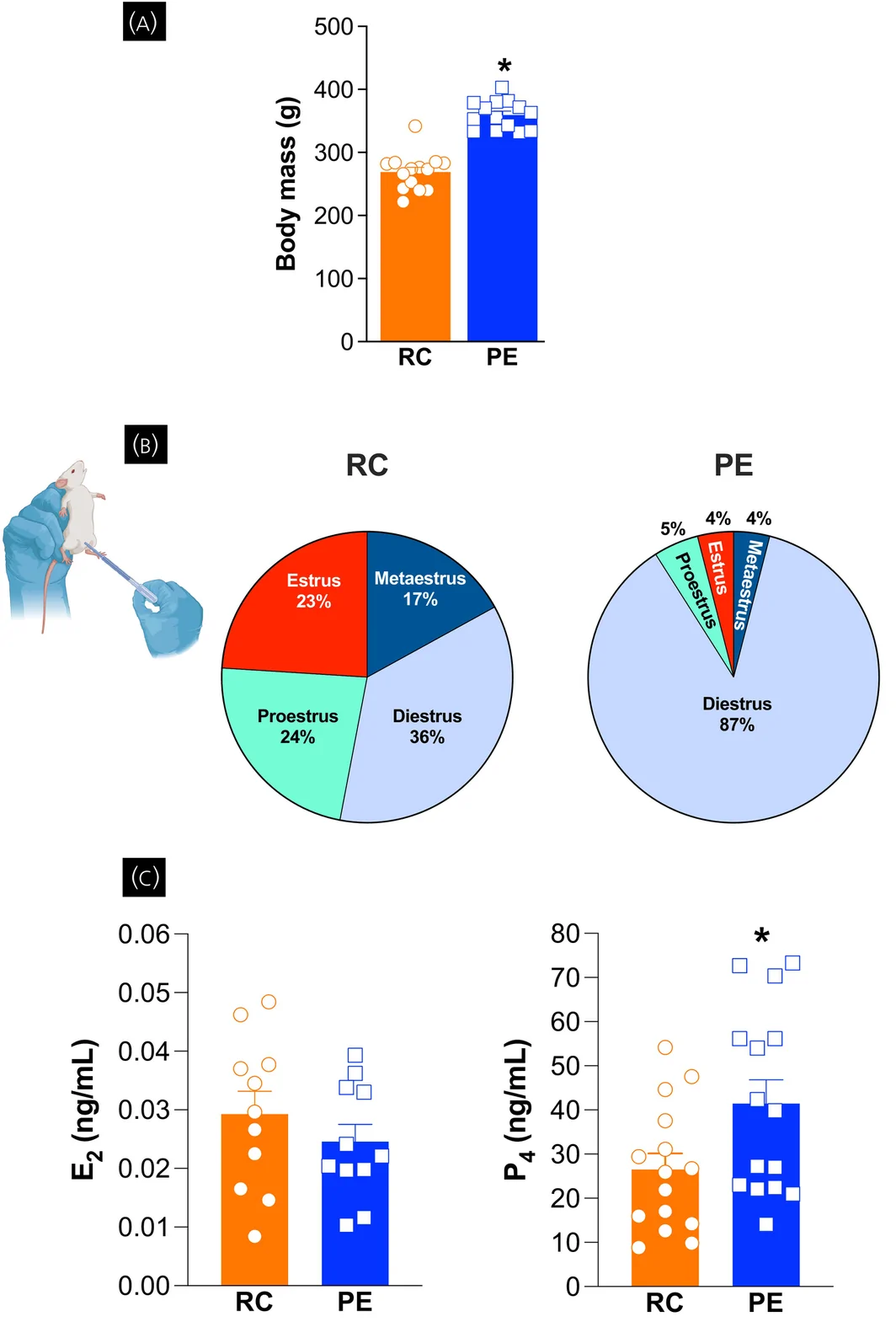
Body mass (A), estrous cycle (B), and plasma steroid hormone concentrations (C) in regularly cycling (RC) and periestropause (PE) *Wistar* rats; (RC: *n* = 11–15; PE: *n* = 11–15). Values are expressed as the mean ± S.D., along with individual values. * Indicates significant differences between groups (unpaired *t*‐test).

PE rats exhibited a significant increase in body mass (*t*_(1,26)_ = 9.21, *p* < .001) throughout the study, whereas RC rats maintained lower body mass values (Figure 2A).

Analysis of the estrous cycle revealed evident alterations in the frequency of occurrence of each phase in the estrous cycle between RC and PE rats (Figure 2B). Specifically, PE rats exhibited a predominance of frequency of diestrus, accounting for 87% of the cycle monitoring period, while RC rats displayed the expected distribution of estrous phases. In PE rats, the frequency of estrus, metestrus, and proestrus was reduced (Figure 2B).

Plasma E_2_ levels were similar in PE and RC rats (Figure 2C). However, plasma P_4_ levels were elevated in PE rats, whereas RC rats exhibited lower concentrations (*t*_(28)_ = 2.294, *p* = .0295) (Figure 2C).

### 3.2 Effect of periestropause on ventilatory and metabolic parameters during the hypercapnic and hypoxic challenge

**FIGURE 3:**
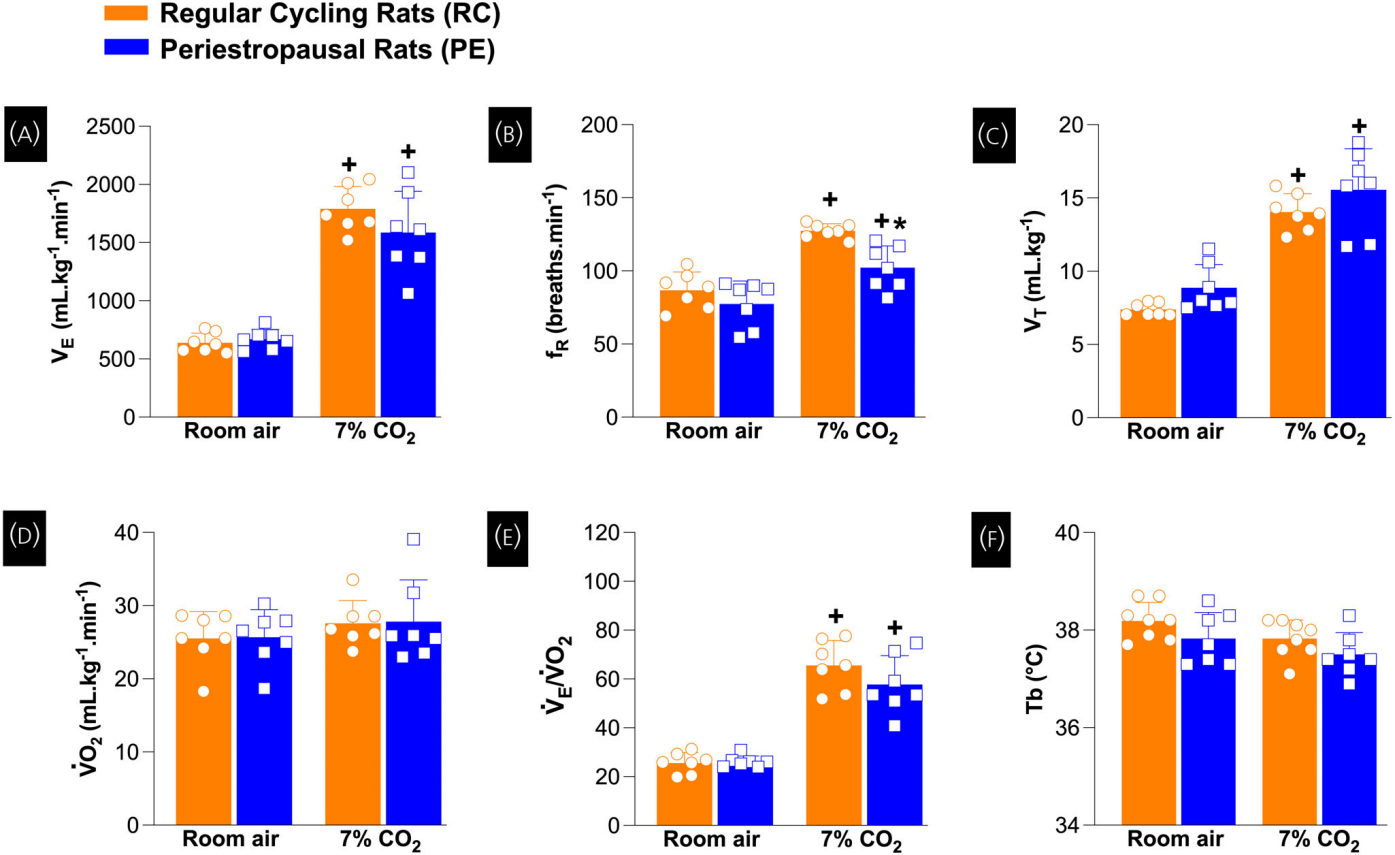
Ventilation (V̇_E_, A), respiratory frequency (*f*_R_, B), tidal volume (*V*_T_, C), oxygen consumption (V̇O_2_, D), and ventilatory equivalent (V̇_E_/V̇O_2_, E) and body temperature (*T*_B_, F) under normocapnic and hypercapnic (7% CO_2_) conditions of regularly cycling (RC) and periestropause (PE) *Wistar* rats; (RC: *n* = 7; PE: *n* = 7). Values are expressed as the mean ± S.D., along with individual values. ^+^Indicates significant difference between gaseous conditions within the same group. * Indicates significant differences between groups within the same gas condition.

Under room‐air conditions, ventilatory and metabolic parameters and Tb were similar in PE and RC rats (Figure 3A–F). Hypercapnia increased V̇_E_ in both groups (*F*_(1,24)_ = 245.88, *p* < .001) (Figure 3A), driven by elevations in *f*_R_ (*F*_(1,24)_ = 46.67, *p* < .001) and *V*_T_ compared to normocapnia (Figure 3B,C). The rise in *f*_R_ during CO_2_ exposure was smaller in PE rats, whereas RC rats exhibited a more pronounced tachypneic response (*F*_(1,24)_ = 13.09, *p* < .0001) (Figure 3B). *V*_T_ increased similarly in PE and RC rats (*F*_(1,24)_ = 102.7, *p* < .0001). Hypercapnia also increased V̇_E_/V̇O_2_ in PE and RC rats (*F*_(1,24)_ = 134.2, *p* < .0001) (Figure 3E) without affecting V̇O_2_ or Tb (Figure 3F).

**FIGURE 4:**
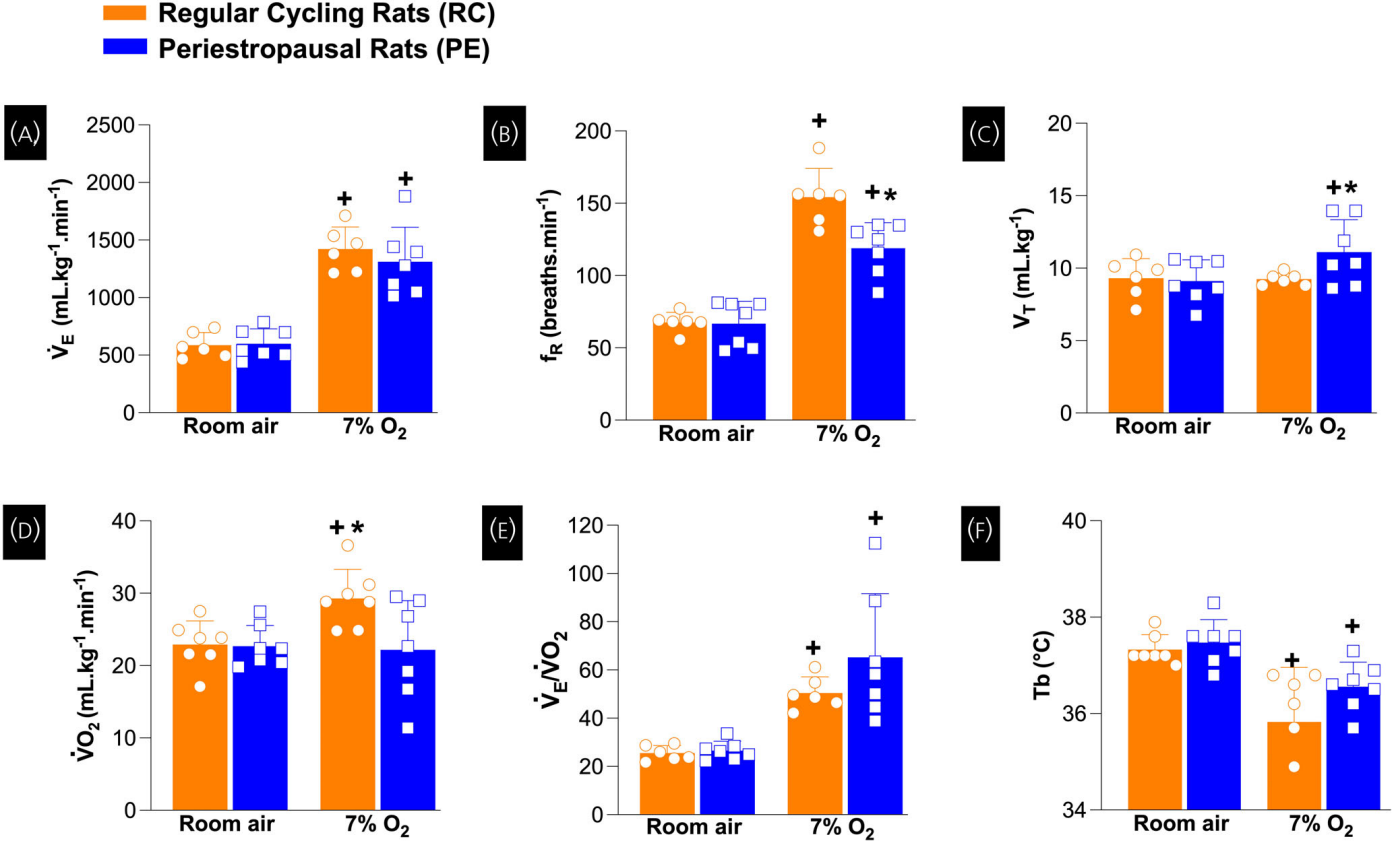
Ventilation (V̇_E_, A), respiratory frequency (*f*_R_, B), tidal volume (*V*_T_, C), oxygen consumption (V̇O_2_, D), and ventilatory equivalent (V̇_E_/V̇O_2_, E) and body temperature (*T*_B_, F) under normoxic and hypoxic (7% O_2_) conditions of regularly cycling (RC) and periestropause (PE) *Wistar* rats; (RC: *n* = 6; PE: *n* = 7). Values are expressed as mean ± S.D. together with individual values. ^+^Indicates significant difference between gaseous conditions within the same group. * Indicates significant differences between groups within the same gas condition.

Hypoxia increased V̇_E_ in RC and PE rats (*F*_(1,22)_ = 96.10, *p* < .0001), primarily driven by an elevation in *f*_R_ (*F*_(1,22)_ = 124.2, *p* < .001) (Figure 4A,B). The increase in *f*_R_ was smaller in PE females, whereas RC rats exhibited a greater tachypneic response (*F*_(1,22)_ = 8.569, *p* = .0081) (Figure 4B). *V*_T_ increased in PE rats (*F*_(1,22)_ = 2.484, *p* = .0251) but differed significantly from the RC group (*F*_(1,22)_ = 1.893, *p* = .0412) (Figure 4C). Hypoxia increased V̇O_2_ in the RC group (*F*_(1,22)_ = 2.978, *p* = .0140), while PE rats exhibited a smaller metabolic response in relation to RC rats (*F*_(1,22)_ = 4.610, *p* = .0421) (Figure 4D). Despite these differences, V̇_E_/V̇O_2_ increased similarly in both groups (*F*_(1,22)_ = 31.65, *p* < .0001) (Figure 4E). Hypoxia‐induced regulated hypothermia was observed in both PE and RC rats (*F*_(1,22)_ = 22.05, *p* < .0001) (Figure 4F).

### 3.3 Effect of periestropause on the hypoxia‐induced panicogenic response

**FIGURE 5:**
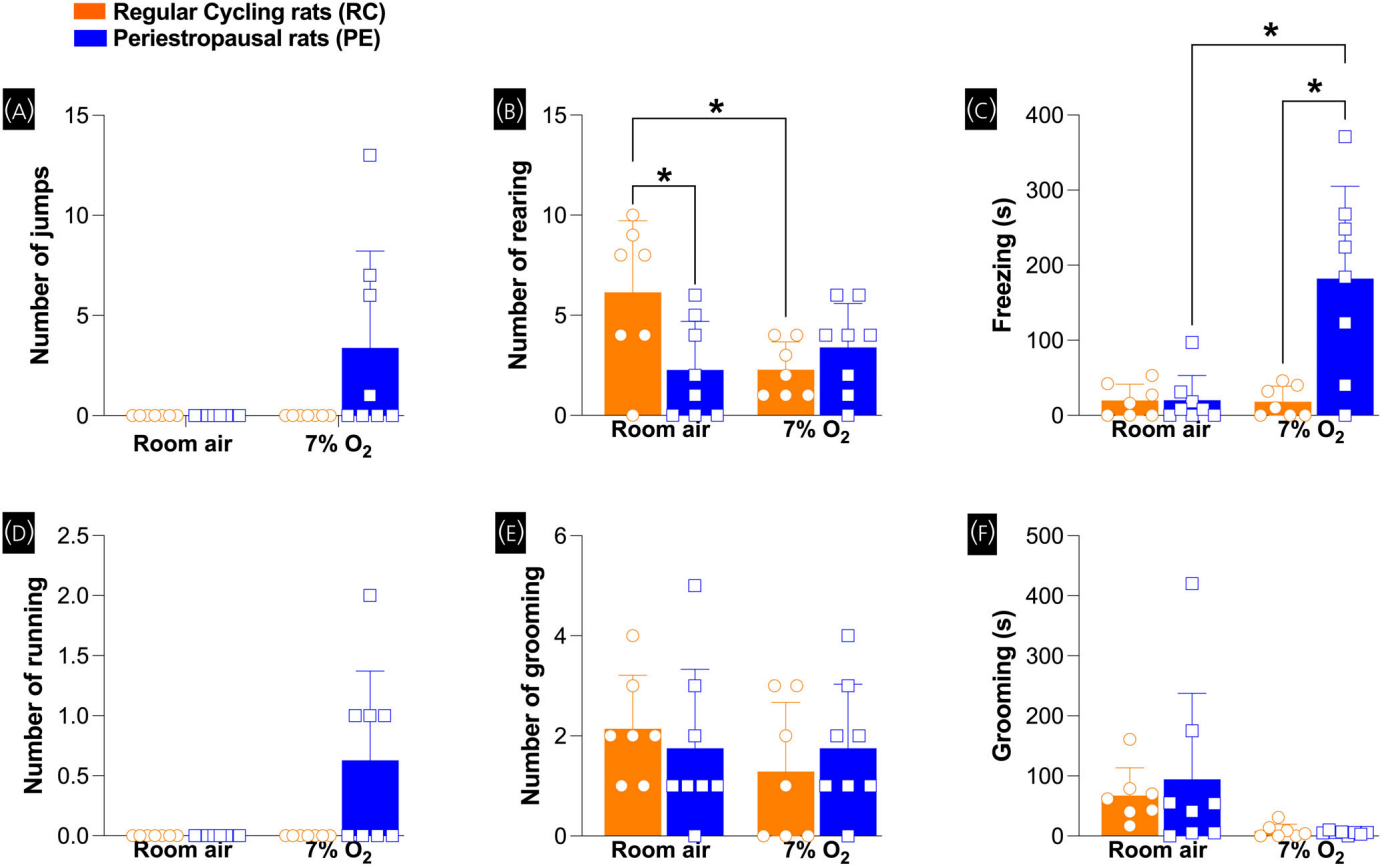
The number of jumps (A), running episodes (B), time in freezing (C), percentage of time in freezing (D), and grooming (E) expressed by regularly cycling (RC) and periestropause (PE) *Wistar* rats subjected to normoxia and 7% O_2_; (RC: *n* = 7; PE: *n* = 8). Behavioural analysis was based on 900 s for both normoxia and hypoxia. Values are expressed as mean ± S.D. A significant main effect of Age was observed for jumping (*p* < .0001) and running (*p* = .012). A significant Age × Gas interaction was detected for freezing (*p < .0001) and rearing (*p = .011), whereas grooming duration showed a significant main effect of Gas (*p* = .002). No significant effects were detected for the number of grooming episodes.

A negative binomial GLM revealed a significant main effect of age on jumping (Wald *χ* ^2^(1) = 33.95, *p* < .0001), whereas neither gas exposure (Wald *χ* ^2^(1) < 0.001, *p* = .9998) nor the Age × Gas interaction (Wald *χ* ^2^(1) < 0.001, *p* = .9998) was significant (Figure 5A).

For running, only a significant main effect of age was detected (Wald *χ* ^2^(1) = 6.286, *p* = .0122) (Figure 5B). Neither gas exposure (Wald *χ* ^2^(1) < 0.001, *p* = .9998) nor the Age × Gas interaction (Wald *χ* ^2^(1) < 0.001, *p* = .9999) was significant.

For freezing, there was no significant main effect of gas exposure (Wald *χ* ^2^(1) = 0.38, *p* = .5397), but there was a significant main effect of age (Wald *χ* ^2^(1) = 1138.38, *p* < .0001) and a significant Age × Gas interaction (Wald *χ* ^2^(1) = 230.94, *p* < .0001) (Figure 5C). Post hoc analysis showed that PE rats exposed to hypoxia exhibited greater freezing behaviour than RC rats exposed to room air or hypoxia, and than PE rats under room air (all *p* < .0001). No differences were observed between RC and PE rats under room air, or between RC under room air and hypoxia.

For rearing, there was a significant main effect of gas exposure (Wald *χ* ^2^(1) = 6.50, *p* = .0108) and a significant Age × Gas interaction (Wald *χ* ^2^(1) = 6.40, *p* = .0114), whereas the main effect of age was not significant (Wald *χ* ^2^(1) = 0.9290, *p* = .335) (Figure 5D). Post hoc analysis revealed that RC under room air displayed more rearing episodes than PE rats under room air (*p* = .041), whereas no other pairwise comparisons were significant.

For the number of grooming episodes, no significant effects of gas exposure (Wald *χ* ^2^(1) = 1.515, *p* = .2182), age (Wald *χ* ^2^(1) = 0.5304, *p* = .4684), or the Age × Gas interaction (Wald *χ* ^2^(1) = 0.822, *p* = .3646) were detected (Figure 5E).

For grooming duration, there was a significant main effect of gas exposure (Wald *χ* ^2^(1) = 9.2948, *p* = .002), whereas neither age (Wald *χ* ^2^(1) = 0.4591, *p* = .4980) nor the Age × Gas interaction (Wald *χ* ^2^(1) = 0.7535, *p* = .3854) was significant (Figure 5F).

### 3.4 Effect of periestropause on the hypoxia‐induced microglia activation

**FIGURE 6:**
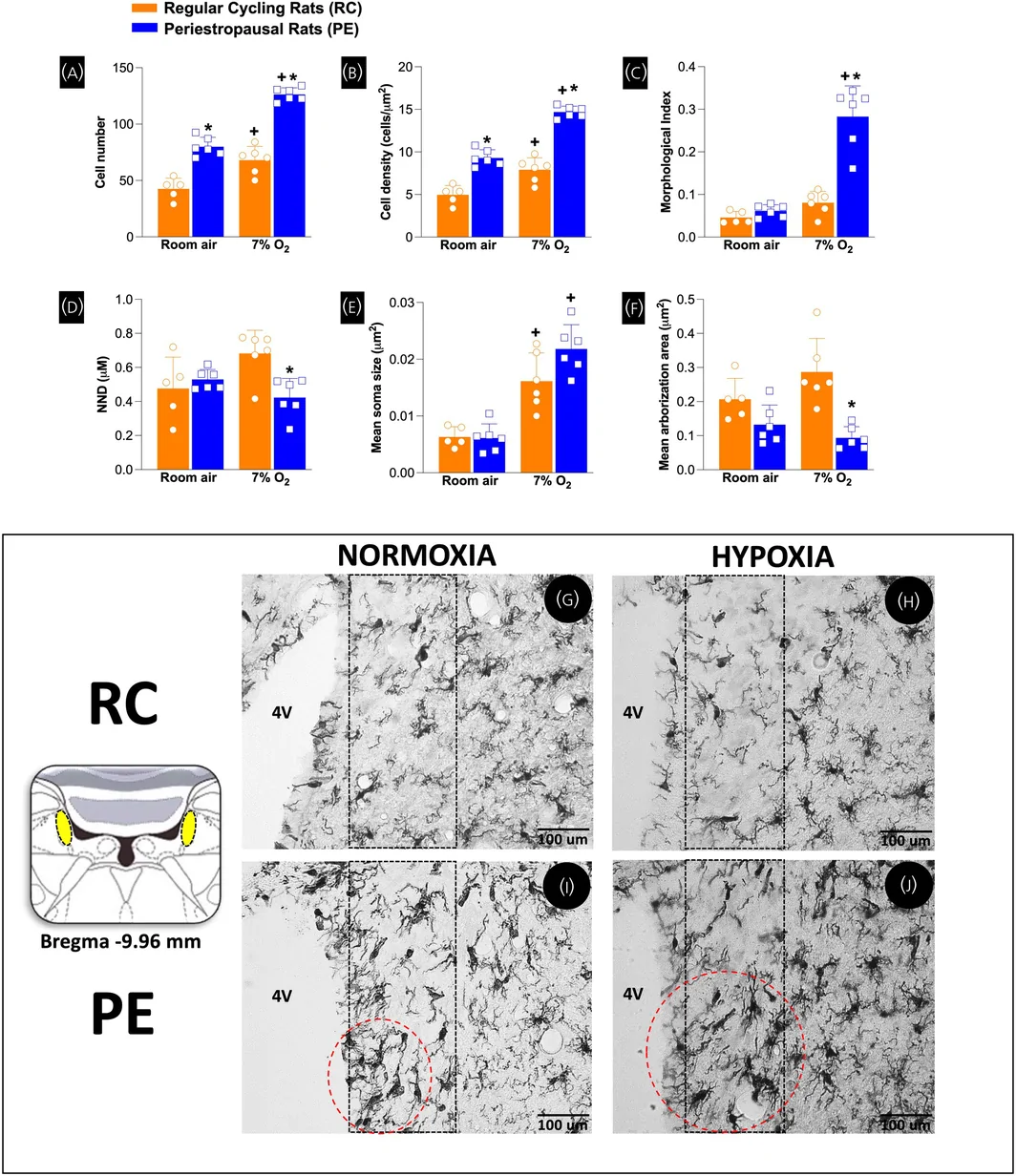
Effect of hypoxia on microglial cells in the LC region (dashed lines). Quantitative analysis of microglial cell parameters 6 h after exposure: Cell number (A), cell density (B), morphological index (C), nearest neighbour distance (NND) (D), mean soma size (E), and mean arborization area (F). Representative Iba‐1 immunohistochemistry in the LC under normoxic and hypoxic conditions from regularly cycling (RC) rats (G, normoxia; H, hypoxia) and periestropause (PE) rats (I, normoxia; J, hypoxia); (RC: *n* = 5; PE: *n* = 6). Scale bar: 100 μm. ^+^Indicates significant differences between gas conditions within the same group; * Indicates significant differences between groups within the same gas condition. The red circle highlights microglial cells within the ‘cluster’.

Under room air conditions, PE females exhibited a higher number of microglial cells and increased microglial cell density in the LC compared with RC rats (*F*_(1,19)_ = 153.5, *p* < .0001) (Figure 6A,B).

**FIGURE 7:**
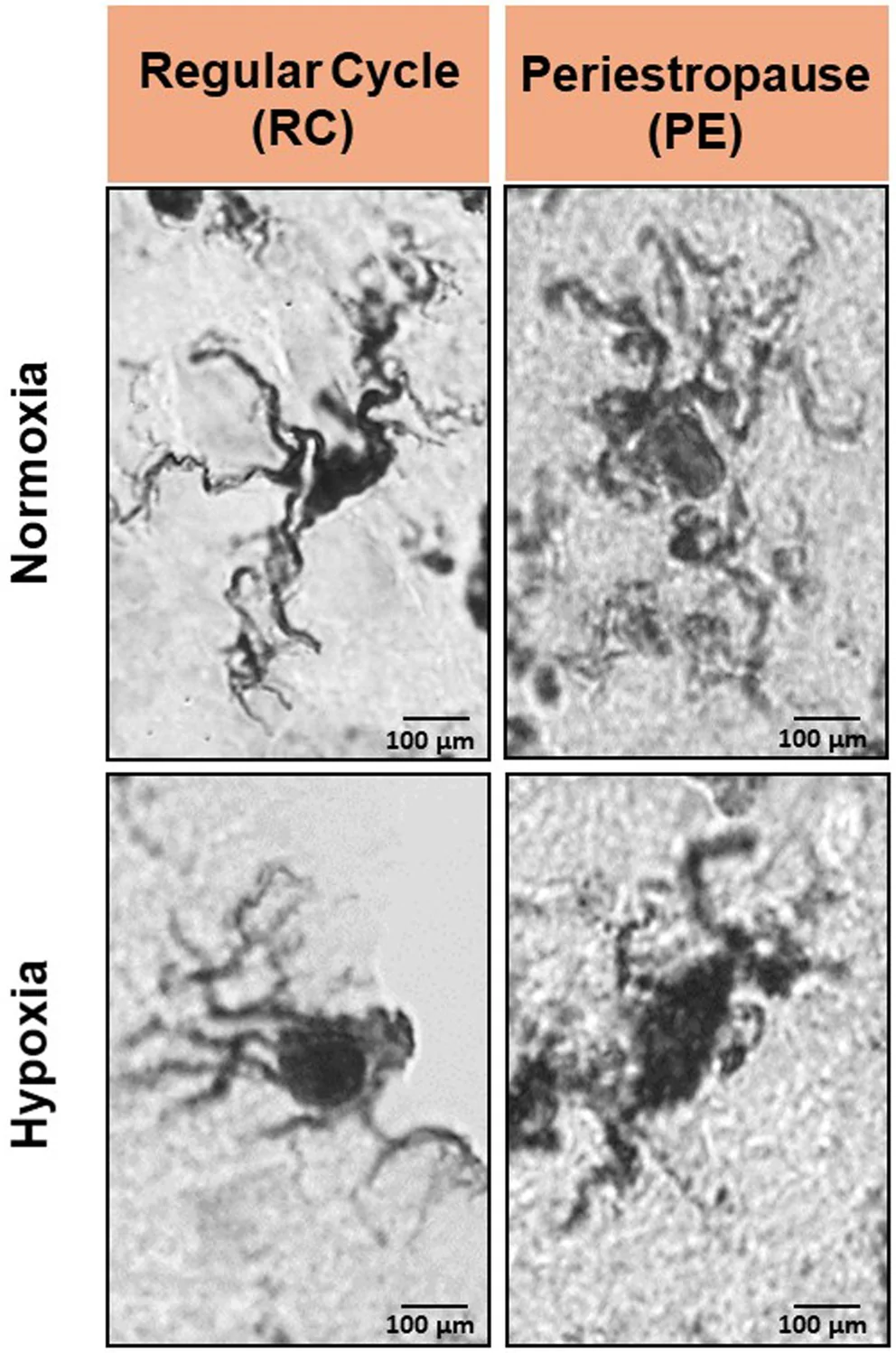
Microglial reactivity in the LC of RC and PE female rats under normoxic and hypoxic conditions. Scale bar: 100 μm.

Exposure to 7% O_2_ increased the number and density of microglial cells in the LC (*F*_(1,19)_ = 86.51, *p* < .0001) in both PE and RC rats (Figure 6A,B), as well as mean soma size (*F*_(1,19)_ = 68.48, *p* < .0001) (Figure 6E). However, these hypoxia‐induced changes were more pronounced in PE females, as indicated by greater increases in the number and density of microglial cells (*F*_(1,19)_ = 7.409, *p* = .0135) (Figure 6A,B). Mean arborization area was reduced in PE females under hypoxia (*F*_(1,19)_ = 4.498, *p* = .0473) (Figure 6F). Hypoxia also increased the morphological index selectively in PE rats (*F*_(1,19)_ = 30.34, *p* < .001; Figure 6C), resulting in higher values than those observed in RC females (*F*_(1,19)_ = 41.40, *p* < .001) (Figure 6C). Regarding the NND, RC females had a higher NND during hypoxia compared with PE rats (*F*_(1,19)_ = 8.55, *p* = .0087) (Figure 6D). Figures 6G–J and 7 show representative immunohistochemistry photomicrographs of Iba‐1 in the LC region under normoxic and hypoxic conditions in RC and PE females. The dashed lines in Figure 6G,H indicate the LC region, and the circle highlights microglial cells within the ‘cluster’ (Figure 6I,J), and Figure 7 shows the 40× magnification of an isolated microglial cell.

### 3.5 Correlation between microglial response and hypoxia‐induced panicogenic behaviour

**FIGURE 8:**
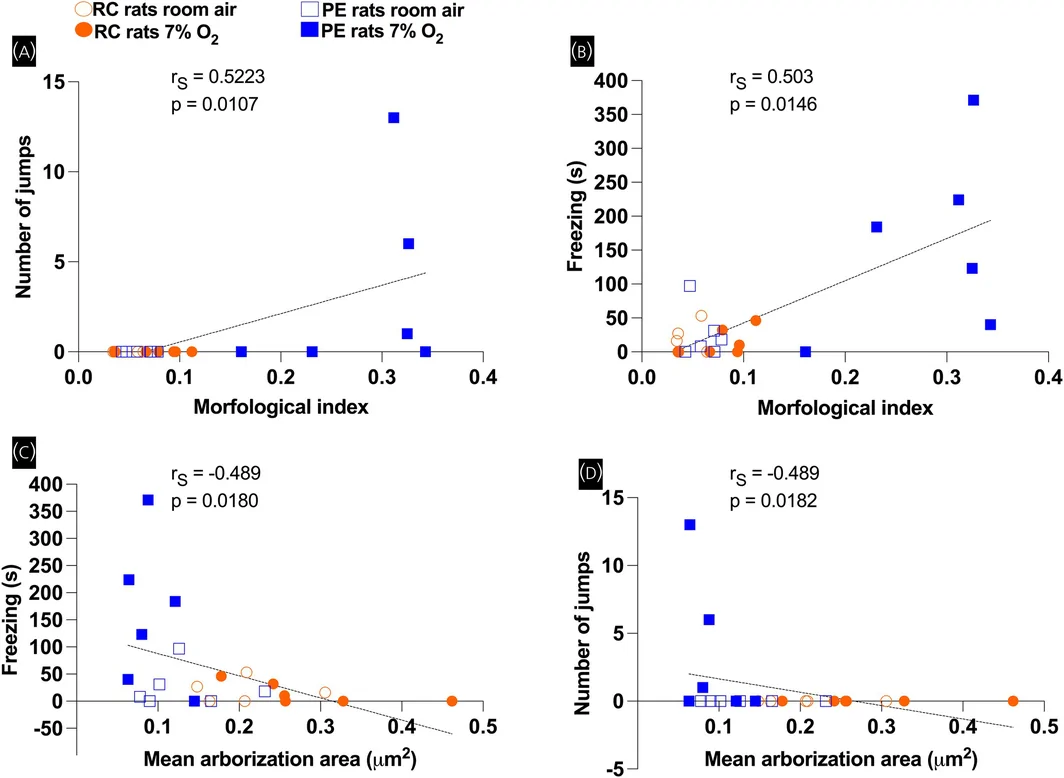
Spearman's correlation between microglial changes and panic‐like behaviour in regular cycling (RC) and periestropausal (PE) rats during normoxia and hypoxia. Plot graphs depicting the number of jumps as a function of morphological index (A) and mean arborization area (C), and freezing duration as a function of morphological index (B) and mean arborization area (D) (RC: *n* = 5; PE: *n* = 6). *r*_S_ denotes the Spearman correlation coefficient, and *p* < .05 was considered statistically significant.

Spearman correlation analysis between jumping behaviour and the microglial morphological index showed a significant positive relationship between these variables (*r*_S_ = 0.5223, *p* = .0107) (Figure 8A). PE rats responded to the hypoxic challenge by increasing the microglial morphological index and the number of jumps. Similarly, the duration of freezing behaviour was also linked to an increase in the microglial morphological index in these animals during hypoxia (*r*_S_ = 0.503, *p* = .0146) (Figure 8B).

An increased number of jumps during exposure to 7% O_2_ was linked to a decrease in the microglial soma arborization area in PE rats (*r*_S_ = −0.489, *p* = .0182). The amount of time spent in freezing behaviour also affected the arborization area, with longer freezing durations during hypoxia significantly reducing microglial arborization in PE animals (*r*_S_ = −0.489, *p* = .0180) (Figure 8C,D).

## 4 DISCUSSION

Periestropause is a well‐recognised transitional stage of female reproductive ageing in rats, typically emerging at approximately 17 months of age. It is characterised by marked changes in ovarian steroid and gonadotropin profiles, together with progressive disruption of estrous cyclicity, primarily a persistent diestrus phase.^5^, ^51^ In the present study, we found that PE female rats had higher body mass compared to RC controls, consistent with metabolic and endocrine changes often associated with reproductive ageing.^5^, ^51^ We also observed significantly elevated plasma P_4_ levels in PE rats, accompanied by E_2_ concentrations similar to those observed in diestrus in rats with a regular estrous cycle.^5^ It is important to highlight that animals with a regular cycle will naturally show a gradual increase in E_2_ concentrations from late diestrus until the occurrence of the preovulatory LH surge in the afternoon of proestrus.

The variability in P_4_ concentrations observed in the present study is consistent with the physiological endocrine fluctuations reported in naturally cycling female rats^52^, ^53^, ^54^ and with the greater inter‐individual variability observed in reproductively aged females.^55^ Direct comparisons of individual hormone values are challenging because most studies report only group summary statistics rather than individual data. Nevertheless, we cannot exclude that individual differences in P_4_ concentrations may have contributed to some variability in the behavioural and respiratory responses, which represents an inherent characteristic of this biological model.

Regarding respiratory responses, this study showed that PE rats exhibited lower respiratory frequency during hypoxic and hypercapnic conditions, yet an increased panicogenic behavioural response during respiratory challenge. Moreover, LC microglial morphology and density were significantly altered under both normoxic and hypoxic conditions, with a higher prevalence of these changes observed in PE rats. These findings suggest an enhanced microglial reactivity within the LC during periestropause, which may reflect a heightened neuroinflammatory state and increased vulnerability of this region to respiratory or metabolic stress.

Ovarian hormones can affect respiratory system activity both centrally and peripherally.^56^ We found no differences in ventilatory or metabolic parameters between RC and PE females under room‐air conditions, suggesting that hormonal changes during ageing are insufficient to alter resting V̇_E_ and V̇O_2_ in rats. Similarly, previous studies from our laboratory showed that the estrous cycle did not influence these parameters in female rats and mice.^37^, ^57^, ^58^ Additionally, a recent study in aged female Fisher rats found no changes in *f*_R_, *V*_T_, inspiratory time, or neural drive under normoxic normocapnic conditions.^59^

Variations in circulating sex hormones, such as those during the normal menstrual cycle in women or the estrous cycle in female rodents, seem to have minimal effects on hypercapnic and hypoxic ventilatory responses.^37^, ^57^, ^60^ During exposure to CO_2_ and hypoxia, similar increases in ventilation were observed in RC and PE females. The only difference was that PE females exhibited a reduced tachypneic component of the ventilatory response. Additionally, PE females had a higher *V*_T_ than RC females. The blunted increase in *f*_R_ in PE females may be linked to hormonal changes related to reproductive ageing, especially the decline in E_2_ and altered P_4_ levels. Since both hypoxic and hypercapnic tachypneic responses were affected, this suggests the effect may involve the carotid body. It is well known that the carotid body has both E_2_ and P_4_ receptors that could influence hypoxic and hypercapnic sensitivity.^61^, ^62^ Unlike the current study, Grittner et al.^59^ found that ageing reduced the hypoxic ventilatory response and the magnitude of ventilatory output to stepwise hypoxia (15% O_2_ to 9% O_2_), primarily due to metabolic changes. Pattern differences in the hypoxic ventilatory responses could be due to differences in rat strain: Fisher^59^ versus Wistar (present study) or hypoxic levels (15% O_2_ to 9% O_2_ –^59^ versus 7% O_2_ – present study).

Besides ventilatory changes, acute hypoxia is also known to trigger panic‐like responses in rats, characterised by escape behaviours and increased anxiety‐like reactions that resemble human panic attacks.^15^, ^16^, ^63^ We observed a decrease in rearing behaviour in PE rats under room‐air conditions, which may indicate reduced exploratory activity and increased anxiety‐like symptoms, likely due to hormonal changes associated with ovarian senescence. Furthermore, RC rats did not exhibit a jumping response during the hypoxic challenge; instead, hypoxic exposure decreased rearing behaviour. The differences between our findings and those of Batistela et al.,^15^ who reported increased jumping responses in females, may be attributable to differences in the hypoxic protocols used in each study. Batistela et al.^15^ induced hypoxia using a rapid N_2_ flush to achieve 7% O_2_, followed by a 6‐min exposure, whereas our protocol employed a slower gas flow with continuous ventilation at 7% O_2_ for 15 min. These differences in the rate of hypoxia onset and exposure duration may influence the magnitude of the stress response and, consequently, behavioural outcomes.

Interestingly, PE females showed more runs and longer freezing times, indicating a stronger panicogenic response. The periestropause period is more sensitive to anxious behaviours like running and freezing, as plasma levels of P_4_ remain elevated during this time,^36^ which positively affects increased anxiety in women during the luteal phase.^64^ Female reproductive cycles are known to influence the progression of panic disorder, often worsening during the menopausal transition.^65^ Among individuals aged 40–49 years, the prevalence of PD is about 3.3%, with women making up 2.4% of cases.^66^

Regarding symptomatology and modelling freezing behaviour, it is widely recognised as a translational indicator of innate fear or panic‐like responses in rodents, closely linked to sympathetic activation and defensive immobility, paralleling key aspects of human panic attack phenotypes.^10^ This freezing behaviour captures a fundamental component of panic‐related immobility and heightened fear that complements other sympathetic symptoms such as agitation, sweating, palpitations, and hyperventilation. Although freezing in animals may not perfectly mimic human reactions, clinical studies show that patients with panic disorder who experience anticipatory anxiety often exhibit reduced mobility, consistent with the freezing phase of the defence cascade.^67^ Thus, increased freezing responses in our rat model can be considered a valid translational model of specific panic attack symptoms involving sympathetic nervous system activation and defensive behavioural patterns.

The reduction in grooming observed after hypoxia exposure in both groups may reflect a behavioural shift from exploratory or self‐maintenance activities toward defensive responses. The animals initially exhibit anticipatory anxiety when placed in the environment,^68^ with no differences between groups. However, when exposed to the panic‐inducing stimulus of hypoxia, the behavioural response of PE females shifts significantly toward adaptive defence mechanisms, including increased flight and freezing behaviours. This change indicates a shift in neural activity from anticipatory anxiety circuits to those specifically activated by immediate physiological threats, highlighting distinct neurobiological pathways involved in responses to hypoxia‐induced panic.^69^

The increase in hypoxic panicogenic response may be connected to heightened activity of the LC region in PE females. A previous study using the same PE model showed that, in acyclic PE female rats, LC noradrenergic neurons exhibit increased activity despite reduced capacity for neurotransmitter synthesis and storage.^5^ This imbalance could impair neuroendocrine signalling needed for luteinizing hormone (LH) release, contributing to ovulatory failure and disrupted hypothalamic–pituitary‐ovarian axis function, a key aspect of reproductive ageing in rodents.

In the current study, we examined LC microglial cells and found that, in PE rats under normoxic conditions, there was a significant increase in both the number and density of microglial cells within the LC. This heightened microglial presence may indicate mild neuroinflammation or increased immune surveillance within this nucleus, even in the absence of respiratory challenges. Given the essential role of LC in regulating arousal,^70^ stress responses,^71^ and neuroendocrine signalling,^24^, ^72^ these microglial changes could contribute to dysregulated noradrenergic activity. This might worsen vulnerability to panic disorder and impair hypothalamic–pituitary‐ovarian axis function, potentially accelerating the neurobiological processes linked to reproductive ageing.

Hypoxia induced significant microglial reactivity in the LC of both RC and PE rats. Specifically, exposure to 7% O_2_ increased the number and density of microglial cells in the LC in both reproductive RC and PE female rats; however, this response was more pronounced in PE animals. This exaggerated microglial reactivity in aged females may reflect an enhanced neuroinflammatory response to hypoxia. Interestingly, under hypoxic conditions in PE rats, there was a significant reduction in arborization area. Thus, the morphological index, as expected, was higher in PE animals. Typically, in the adult mouse brain under noninflammatory conditions, microglia have a small cell body with highly ramified branches that constantly survey the cerebral parenchyma.^73^ In our study, the reduction in arborization area indicates that microglia are reactive and retract their branches, adopting an amoeboid morphology. These morphological changes reflect a less complex structure, which is associated with increased phagocytosis capacity.^74^ Indeed, it has been demonstrated that reactive/amoeboid microglia are the most prevalent population in the aged human brain.^75^ Finally, the NND analysis revealed a lower NND index in PE females under hypoxia (Figure 6D). The lower NND index indicates that microglial cells are closer to each other (‘clustered’ – Figure 6 I,J); in other words, microglia are moving to or have already moved to the LC in response to hypoxia. This explains why the NND is considered an index of motility.^48^ Thus, as microglia are closer to other cells, a higher cellular density is expected (Figure 6B). Another possibility that could explain the higher density observed in PE rats during normoxia and hypoxia compared with RC rats is that, when microglial cells become reactive and exhibit increased motility, they undergo proliferation.^76^ However, this response usually takes days to happen, and as we see increased cell density even during normoxia, the ageing process and the hormonal imbalance ongoing in PE rats could be the “first” factor that activates microglia, followed by the acute stress (hypoxia), which causes even a stronger activation in those animals.

The delayed microglial activation observed 6 h after hypoxic exposure aligns with our previous findings, which show that microglial activation is absent at 1 h and returns to baseline by 24 h after a similar acute hypoxic challenge.^77^ Likewise, previous studies have shown that morphological microglial activation becomes evident only several hours after acute hypoxic or brain injury stimuli, reflecting the time required for the transition from a ramified to an activated phenotype.^78^, ^79^, ^80^ Such neuroimmune signalling could facilitate short‐term plasticity within LC circuits that regulate respiratory and stress‐related responses. However, Tadmouri et al.^80^ reported that a single brief hypoxic challenge (3–5 min at 10% O_2_/90% N_2_) induced microglial activation in the nucleus of the solitary tract, which persisted for up to 24 h. Whether repeated hypoxic episodes lead to sustained microglial activation and long‐term alterations in LC function warrants further investigation.

There is a significant correlation between microglial morphology and hypoxia‐induced panic‐like behaviour. The correlation analyses further indicate that this relationship is not uniform across groups, with the effect predominantly driven by PE females exposed to hypoxia (Figure 8). These animals account for most of the variability and strongly influence the observed associations, with higher morphological indices and reduced arborization areas correlating with increased freezing and escape behaviours. In contrast, RC and normoxic groups show limited variability and contribute minimally to these relationships. This pattern suggests that the observed microglial changes are not merely a consequence of periestropause but are more closely linked to the response to a panicogenic challenge. Therefore, although an age effect cannot be entirely excluded, our data do not support it as the primary driver. Instead, they indicate that microglial remodelling is specifically associated with the heightened vulnerability of PE animals to hypoxia‐induced panic‐like responses, underscoring an interaction between periestropause and an acute environmental stressor.

Sex steroids cross the blood–brain barrier and, through receptor‐mediated mechanisms, exert anti‐inflammatory and neuroprotective effects on microglial cells.^34^, ^81^, ^82^ However, these actions may be compromised by age‐related increases in microglial neuroinflammation^83^, ^84^ and by a decline in microglial phagocytic capacity.^85^ In this context, elevated P_4_ levels observed in PE females are associated with increased microglial reactivity and may reflect a diminished anti‐inflammatory capacity. Moreover, the anxiolytic effects of progesterone (P_4_) depend on its neuroactive metabolite, 3α,5α‐tetrahydroprogesterone (allopregnanolone).^86^, ^87^ Although this metabolite was not measured in the present study, future studies should determine whether its levels, or the activity of the enzymes responsible for its synthesis, are altered in PE animals, as impaired conversion of P_4_ to allopregnanolone could contribute to the enhanced anxiety‐like behaviour observed during panic induction. Overall, these findings suggest that reproductive ageing is associated with altered microglial plasticity in the LC, which may impair central chemosensory processing and contribute to dysfunctional panicogenic behavioural responses in aged females.

A limitation of the current study is the lack of age‐matched control groups, which makes it difficult to clearly distinguish the effects of ageing, strain, and reproductive hormonal status on the observed ventilatory and behavioural responses. Consequently, our results should be seen as reflecting the combined influence of reproductive senescence and ageing, rather than a direct causal effect of female hormones. In addition to changes in reproductive hormone levels, ageing is associated with a range of physiological alterations, including changes in cognition,^88^ locomotor performance,^89^ oxidative stress,^89^ bone remodelling,^90^ and noradrenergic neurotransmission,^5^ all of which may have contributed to the responses observed in the present study. Future research using age‐matched groups and experimental methods such as direct hormonal manipulation (e.g., ovariectomy and hormone replacement) will be crucial to identify the specific role of sex hormones in respiratory and panic‐related responses.

Taken together, our study showed that although respiratory responses change only mildly, with slight decreases in respiratory frequency during hypoxic and hypercapnic challenges, PE female rats exhibit increased vulnerability to panic‐inducing hypoxia and signs of neuroinflammation. Notably, increased microglial activation was observed in the LC under both normal oxygen conditions and during hypoxia, suggesting a chronic, low‐grade neuroinflammatory state. These findings indicate that although basic respiratory regulation remains largely preserved during reproductive ageing, PE rats display increased behavioural sensitivity to hypoxia and enhanced neuroimmune activation within the LC. Neuroimmune changes and increased emotional reactivity may elevate the risk of panic and stress‐related disorders in ageing females.

## AUTHOR CONTRIBUTIONS

**Angela Cristina Nicola:** Conceptualization; investigation; methodology; validation; writing – original draft; visualization; writing – review and editing; formal analysis; data curation. **Luana Tenório‐Lopes:** Investigation; methodology; visualization; writing – review and editing; formal analysis; writing – original draft. **Luis Gustavo A. Patrone:** Investigation; methodology; writing – review and editing; visualization; formal analysis. **Mariana Bernardes‐Ribeiro:** Methodology; writing – review and editing; formal analysis; validation; visualization. **Heloisa H. Vilela Costa:** Methodology; validation; writing ‐ review and editing; formal analysis. **Daniele Gandra:** Validation; methodology; writing – review and editing; visualization; formal analysis. **Ana Clara Campideli‐Santana:** Methodology; validation; visualization; writing – review and editing; formal analysis. **Raphael E. Szawka:** Methodology; validation; visualization; writing – review and editing; formal analysis. **Kênia C. Bícego:** Investigation; visualization; writing – review and editing. **Rita Cássia M. Dornelles:** Investigation; conceptualization; validation; visualization; writing – review and editing; supervision. **Luciane H. Gargaglioni:** Conceptualization; investigation; funding acquisition; writing – original draft; methodology; validation; visualization; writing – review and editing; project administration; data curation; supervision; resources.

## CONFLICT OF INTEREST STATEMENT

The authors declare no conflicts of interest.

## ETHICS STATEMENT

All experiments with mice were conducted with the approval of the local College of Agricultural and Veterinary Sciences Animal Care and Use Committee (CEUA‐FCAV‐UNESP‐Jaboticabal; Protocol n° 11.794).

## Supporting information


**Table S1:** Results of unpaired *t*‐test statistical analyses for body mass, estrogen (E_2_) and progesterone (P_4_) concentration in regular cycling (RC) and periestropause (PE) females.
**Table S2:** Results of two‐way ANOVA statistical analyses for ventilatory and metabolic parameters in regular cycling (RC) and periestropause (PE) females during normocapnia and hypercapnia.
**Table S3:** Results of two‐way ANOVA statistical analyses for ventilatory and metabolic parameters in regular cycling (RC) and periestropause (PE) females during normoxia and hypoxia.
**Table S4:** Results of two‐way ANOVA statistical analyses for behaviour of regular cycling (RC) and perimenopause (PE) females during normoxia or hypoxia.
**Table S5:** Results of two‐way ANOVA statistical analyses for microglia in the LC in regular cycling (RC) and perimenopausal (PE) females during normoxia or hypoxia.

## Data Availability

The data that support the findings of this study are available from the corresponding author upon reasonable request.
