# Supplementary material for: Periestropause enhances hypoxia‐induced panic and microglial remodelling without altering respiratory control
Source: J Neuroendocrinol. 2026 Sep 3;38(9):e70254. doi: 10.1111/jne.70254 (PMC13539373; doi:10.1111/jne.70254)
Supplement: Supplementary file 1 [file JNE-38-e70254-s001.docx]

**Table S1:** Results of unpaired t-test statistical analyses for body mass, estrogen (E_2_) and progesterone (P_4_) concentration in regular cycling (RC) and periestropause (PE) females.

|  | **UNPAIRED T TEST** | | |
| --- | --- | --- | --- |
|  | **Body mass** | **E_2_** | **P_4_** |
| RC x PE | t_(26)_ = 9.21  p < 0.0001***** | t_(20)_ = 0.962  p = 0.348 | t_(28)_ = 2.29  p = 0.0295***** |

**Table S2:** Results of two-way ANOVA statistical analyses for ventilatory and metabolic parameters in regular cycling (RC) and periestropause (PE) females during normocapnia and hypercapnia.

| **HYPERCAPNIA** | | | | | | |
| --- | --- | --- | --- | --- | --- | --- |
| **TWO-WAY ANOVA** | **Ventilatory and metabolic parameters – TWO-WAY ANOVA REPEATED MEASURES** | | | | | |
|  | **V_E_** | **V_T_** | **f_R_** | **VO_2_** | **V_E_/VO_2_** | **Tb** |
| Exposure effect | F_(1,24)_ = 170.0  p < 0.0001***** | F_(1,24)_ = 102.7  p < 0.0001***** | F_(1,24)_ = 46.67  p < 0.0001***** | F_(1,24)_ = 1.1762  p = 0.1968 | F_(1,24)_ = 134.2  p < 0.0001***** | F_(1,26)_ = 4.694  p = 0.0396***** |
| Age effect | F_(1,24)_ = 1.198  p = 0.2846 | F_(1,24)_ = 5.92  p = 0.0319***** | F_(1,24)_ = 13.09  p = 0.0014***** | F_(1,24)_ = 1.1762  p = 0.1968 | F_(1,24)_ = 1.1762  p = 0.1968 | F_(1,26)_ = 4.597  p = 0.1968 |
| Exposure and Age interaction | F_(1,24)_ = 2.146  p = 0.11559 | F_(1,24)_ = 0.00120  p = 0.9726 | F_(1,24)_ = 12.713  p = 0.1126 | F_(1,24)_ = 0.01268  p = 0.9113 | F_(1,24)_ = 1.413  p = 0.2462 | F_(1,26)_ = 0.01131  p = 0.0415***** |

**Table S3:** Results of two-way ANOVA statistical analyses for ventilatory and metabolic parameters in regular cycling (RC) and periestropause (PE) females during normoxia and hypoxia.

| **HYPOXIA** | | | | | | |
| --- | --- | --- | --- | --- | --- | --- |
| **TWO-WAY ANOVA** | **Ventilatory and metabolic parameters – TWO-WAY ANOVA REPEATED MEASURES** | | | | | |
|  | **V_E_** | **V_T_** | **f_R_** | **VO_2_** | **V_E_/VO_2_** | **Tb** |
| Exposure effect | F_(1,22)_ = 96.10  p < 0.0001***** | F_(1,22)_ = 2.484  p = 0.1293 | F_(1,22)_ = 124.2  p < 0.0001 ***** | F_(1,22)_ = 2.978  p = 0.0992 | F_(1,22)_ = 31.65  p < 0.0001 ***** | F_(1,22)_ = 22.05  p < 0.0001 ***** |
| Age effect | F_(1,22)_ = 0.3691  p = 0.5356 | F_(1,22)_ = 1.893  p = 0.1827 | F_(1,22)_ = 8.469  p = 0.0081***** | F_(1,22)_ = 4.610  p = 0.0421***** | F_(1,22)_ = 1.967  p = 0.1748 | F_(1,22)_ = 2.873  p = 0.1030 |
| Exposure and Age interaction | F_(1,22)_ = 0.6177  p = 0.4403 | F_(1,22)_ = 2.859  p = 0.1050 | F_(1,22)_ = 7.687  p = 0.0111 | F_(1,22)_ = 4.096  p = 0.0543 | F_(1,22)_ = 1.471  p = 0.2381 | F_(1,22)_ = 1.298  p = 0.2658 |

**Table S4:** Results of two-way ANOVA statistical analyses for behaviour of regular cycling (RC) and perimenopause (PE) females during normoxia or hypoxia.

| **BEHAVIOUR - generalized linear model (GLM) with a negative binomial distribution** | | | | | |  |
| --- | --- | --- | --- | --- | --- | --- |
|  | **Jumping** | **Running** | **Freezing** | **Rearing** | **Number of**  **Grooming** | **Grooming** |
| Exposure effect | χ²(1) = 0  p = 0.9998 | χ²(1) = 0  p = 0.9998 | χ²(1) = 0.38  p = 0.5397 | χ²(1) = 6.50  p = 0.0108***** | χ²(1) = 1.515  p = 0.2182 | χ²(1) = 9.2948  p = 0.0023***** |
| Age | χ²(1) = 33.95  p < 0.0001***** | χ²(1) = 6.286  p = 0.0122***** | χ²(1) = 1138.38  p < 0.0001***** | χ²(1) = 0.9290  p = 0.335 | χ²(1) = 0.5304  p = 0.4684 | χ²(1) = 0.4591  p = 0.4980 |
| Exposure and Age interaction | χ²(1) = 0  p = 0.9998 | χ²(1) = 0  p = 0.9998 | χ²(1) = 230.94  p < 0.0001***** | χ²(1) = 6.40  p = 0.0114***** | χ²(1) = 0.822  p = 0.3646 | χ²(1) = 0.7535  p = 0.3854 |

**Table S5:** Results of two-way ANOVA statistical analyses for microglia in the LC in regular cycling (RC) and perimenopausal (PE) females during normoxia or hypoxia.

|  | **Microglia analysis – TWO-WAY ANOVA NON REPEATED MEASURES** | | | | | |
| --- | --- | --- | --- | --- | --- | --- |
|  | **Cell Number** | **Cell density** | **Morphological index** | **NDD** | **Cell body area** | **Arborization area** |
| Exposure effect | F_(1,19)_ = 86.51  p < 0.0001***** | F_(1,19)_ = 86.51  p < 0.0001***** | F_(1,19)_ = 57.25  p < 0.0001***** | F_(1,19)_ = 0.8672  p = 0.3634 | F_(1,19)_ = 68.48  p < 0.0001***** | F_(1,19)_ = 0.5575  p = 0.4644 |
| Age | F_(1,19)_ = 153.5  p < 0.0001***** | F_(1,19)_ = 153.5  p < 0.0001***** | F_(1,19)_ = 41.40  p < 0.0001***** | F_(1,19)_ = 3.743  p = 0.0680 | F_(1,19)_ = 3.156  p = 0.0917 | F_(1,19)_ = 22.87  p = 0.0001 ***** |
| Exposure and Age interaction | F_(1,19)_ = 7.409  p = 0.0135***** | F_(1,19)_ = 7.409  p = 0.0135***** | F_(1,19)_ = 30.34  p < 0.0001***** | F_(1,19)_ = 8.55  p = 0.0087***** | F_(1,19)_ = 3.651  p = 0.0712 | F_(1,19)_ = 4.498  p = 0.0473 ***** |
